# Correlation between the initial CT chest findings and short-term prognosis in Egyptian patients with COVID-19 pneumonia

**DOI:** 10.1186/s43055-021-00685-w

**Published:** 2022-01-04

**Authors:** Mohamed Mohamed Hefeda, Dalia Ezzat Elsharawy, Tamer Mahmoud Dawoud

**Affiliations:** 1 Radiology Department, Faculty of Medicine, Tanta University, Tanta, Egypt; 2 Faculty of Medicine, Tanta University, Tanta, Egypt

**Keywords:** COVOD-19, COVID-19 chest CT, COVID-19 pneumonia, COVID-19 prognosis, COVID-19 CT severity score

## Abstract

**Background:**

The recent pandemic of COVID‐19 has thrown the world into chaos due to its high rate of transmissions. This study aimed to highlight the encountered CT findings in 910 patients with COVID-19 pneumonia in Egypt including the mean severity score and also correlation between the initial CT finding and the short-term prognosis in 320 patients.

**Results:**

All patients had confirmed COVID-19 infection. Non-contrast CT chest was performed for all cases; in addition, the correlation between each CT finding and disease severity or the short-term prognosis was reported. The mean age was higher for patients with unfavorable prognosis (*P* < 0.01). The patchy pattern was the most common, found in 532/910 patients (58.4%), the nodular pattern was the least common 123/910 (13.5%). The diffuse pattern was reported in 124 (13.6%). The ground glass density was the most common reported density in the study 512/910 (56.2%). The crazy pavement sign was reported more frequently in patients required hospitalization or ICU and was reported in 53 (56.9%) of patients required hospitalization and in 29 (40.2%) patients needed ICU, and it was reported in 11 (39.2%) deceased patients. Air bronchogram was reported more frequently in patients with poor prognosis than patients with good prognosis (16/100; 26% Vs 12/220; 5.4%). The mean CT severity score for patients with poor prognosis was 15.2. The mean CT severity score for patients with good prognosis 8.7., with statistically significant difference (*P* = 0.001).

**Conclusion:**

Our results confirm the important role of the initial CT findings in the prediction of clinical outcome and short-term prognosis. Some signs like subpleural lines, halo sign, reversed halo sign and nodular shape of the lesions predict mild disease and favorable prognosis. The crazy paving sign, dense vessel sign, consolidation, diffuse shape and high severity score predict more severe disease and probably warrant early hospitalization. The high severity score is most important in prediction of unfavorable prognosis. The nodular shape of the lesions is the most important predictor of good prognosis.

## Introduction

On December 31, 2019, China reported to the WHO the appearance of respiratory illness in a cluster of people in Wuhan city. On January 12, 2020, the WHO confirmed that a novel corona virus was the cause of the illness [1]. In Egypt, the first case was announced February 12, 2020 and second case on 1 March. As of December 3, 117,158 confirmed cases with 5.7% mortality rate in all Egyptian territories [2].

Though the microbiological tests especially reverse-transcriptase polymerase chain reaction (RT-PCR) are considered the gold standard test for diagnosis of COVID-19 infection, yet, the sensitivity of the RT-PCR is not perfect (only about 89%), and negative result do not exclude COVID-19 pneumonia [3, 4]. Also, false positive results were reported owing to technical errors [5].

From the start of the pandemic, CT has been used as complementary to RT-PCR in the diagnosis and management of COVID-19 pneumonia. Numerous reports and studies described the CT findings of COVID-19 pneumonia. The typical CT findings included ground glass opacities, consolidation, vascular enlargement, air bronchogram, crazy pavement sign, halo signs and reversed halo sign [6–21], with the distribution of the abnormalities was mostly bilateral, in the lower lobes, peripheral and sub-pleural [22]. Most reports described CT chest findings in patients from China or Europe and only few reports described CT chest finding in African countries [23–27]. It is unclear if the disease has the same CT characteristics as in different countries.

The prediction of mortality in COVID-19 pneumonia has relied on demographic and clinical factors like patient’s age and associated co-morbidities [28, 29]. Identification of certain signs on initial CT chest examination may help to predict the prognosis and mortality in even young and healthy patients and may aid to detect the most vulnerable patients and consequently may change the treatment protocol and lines of management. Multiple studies have suggested different prognostic signs [30–33]. A semi-quantitative scoring system of lung involvement on CT has been suggested as useful marker in differentiation between severe illness group and other groups and is referred to as total severity score (TSS) [34].

The aim of this study was to describe the CT findings in Egyptian patients with confirmed COVID-19 pneumonia, and correlation between the initial chest CT findings and the CT severity score with short-term prognosis.

## Methods

### Patients

This retrospective study was approved by the institutional review board, and the informed consent was waived because of the outbreak of COVID-19.

The study included 910 consecutive patients, who were suspected to have COVID 19 pneumonia, in the time interval March 2, 2020, to November 18, 2020. CT beside laboratory test was used as screening tests for COVID 19 pneumonia. All patients included in the study subsequently performed reverse-transcription polymerase chain reaction (RT-PCR) in nasal-pharyngeal swabs and were found to be positive. After one month we could follow 320 patients either by phone, direct contact or re-scanning for correlation of the initial CT findings with the clinical course. If the patient had multiple CT scans, the CT scan interpreted was only the initial CT scan. The time interval from the initial symptoms was from 1 to 8 days. We excluded all patients with initially normal CT scan and patients with all co-morbidities were included in the study.

Clinically, patients were categorized into 4 groups: Patients with mild symptoms who treated at home, stable patients treated in hospitals, critical patients needed ICU, and patients who died. The first two groups are considered good prognosis and the other two groups are considered poor prognosis.

### High-resolution CT imaging

All high-resolution CT scans were obtained using SOMATOM Emotion 16 slices (Siemens, Germany). Images were acquired using following parameters: 2–4 mm section thickness, with axial reconstruction in 1 mm thickness; 20–40 mAs; 120–150 kV. The collimation width was 0.7 mm. The acquisition range was from thoracic inlet to below the diaphragm by 5 cm. Multi-plane reconstruction (MPR) in sagittal and coronal planes technology was performed with a slice thickness of 2 mm. The patients were laid supine, with the arms extended and if possible they were asked to hold breath. The recommended infection prevention and control measures were taken in all cases, including prompt sanitation of CT facility and the entire room.

The images were assessed with window width 1200–2000 HU, 500–700 HU Window level for lung window and window width 300–400 /window level 30–40 HU for mediastinal window.

### CT chest imaging interpretation

All the CT images of all patients were analyzed by two radiologists with 20 and 10 years of chest CT experience independently, blinded to the clinical data, and laboratory indicators, in a standard clinical picture Archiving and Diagnostic System workstation, and final decisions reached by consensus are reported.

The following CT features were assessed: distribution pattern: unilateral or bilateral, number of lobes involved (one, two, or more); the density of the lesions (ground-glass opacity [GGO], consolidation, or GGO with consolidation); shape of the lesions: diffuse, patchy, nodular or mixed. Different signs: crazy paving pattern (ground glass opacity with superimposed interlobular septal thickening), sub-pleural transverse lines, halo sign (circular area of ground glass attenuation surrounding pulmonary nodule), reversed halo sign (central area of ground glass opacity surrounded by area of consolidation), large vessel sign (vessel diameter larger than expected), dense vessel sign (vessels denser than expected in non-contrast study), mediastinal lymph node enlargement, and pleural effusion.

### CT severity score (CT-SS)

In the current study, we adopted the *semi-quantitative scoring system* proposed by Pan et al. [35] was used to assess volume of lung involved. Each of the five lung lobes was subjectively scored on a scale of 0–5, with 0 indicating no involvement, 1 equals less than 5% involvement, 2 when 5–25% involvement, 3 when 26–49% involvement, 4 when 50–75% involvement, and 5 indicating more than 75% involvement. The total CT score was the sum of the individual lobar scores and ranged from 0 (no involvement) to 25 (maximum involvement).

Mild severity when the score is 1–8. Moderate severity score 9–17, severe disease 18–25 score.

### Statistical analyses

Statistical analysis was performed with SPSS (Version 23.0, IBM Corp., USA). Categorical variables are expressed as number percentages. The diagnosis accuracy between HRCT features combined with epidemiological history and the first detection of viral nucleic acid was compared using χ 2 test or Fisher’s exact test. A *P* value < 0.05 was considered statistically significant.

### Results

### Patient demographics, clinical and laboratory findings

The patient's clinical and laboratory findings and the associated Co-morbidities are summarized in Table 1. The study included 910 patients (median age 55 years; range 19–90 years old), 582 (58.03) were males and 382 (41.97%) were females. We could follow the short-term prognosis of 320 patients. The correlation between the clinical data and the short-term prognosis is summarized in Table 1.

**Table 1:** Demographic data in all 910 patients with COVID-19 pneumonia and correlation with prognosis in 320 patients

	All patients (910)	Stable patients (127/320)	Hospitalized stable patients (93/320)	Patients needed ICU (72/320)	Died (28/320)	*P* value
Age						
Median	55.2	36.5	42.4	51.4	57.7	< 0.01
Range	(19–90)	(19–72)	(34–71)	(40–75)	38–87	
Gender						
Male	528(58.02%)	61 (48.03%)	57 (61.29%)	38 (52.77%)	17(60.71%)	> 0.05
Female	382 (41.97%)	65 (51.18%)	36 (38.79%)	34 (47.22%)	11(39.28%)	
Clinical						
Fever	802 (88.13%)	102(80.03%)	87 (93.54%)	68 (94.44%)	28 (100%)	0.651
Cough	765 (84.06%)	97(76.3%)	65 (69.89%)	65 (90.27%)	26 (92.85%)	0.45
Dyspnea	412 (45.27%)	19(14.96)	56 (60.21%)	54 (75.00%)	28(100%)	< 0.05
Sore throat	350 (38.46%)	28(22.04%)	38 (40.86%)	23(31.94%)	11(39.28%)	0.541
GIT symptoms	137 (15.05%)	35(27.55%)	23 (24.73%)	18 (25.00%)	8 (28.57%)	0.432
Muscle pain	522 (57.36%)	58 (45.66%)	54 (58.06%)	61 (84.72%)	22 (78.5%)	0.58
Laboratory investigations						
SP O2 (%)	91 (62–99)	93 (80–99)	88 (75–98)	72 (80–99)	69 (62–81)	0.01
Lymphocytes (%)	14.8% (3.2–49.5)	15.2(6–49.5)	12.2(4.4–45.5)	10.2(5.3–40.5)	8.2(3.2–11.45)	0.07
CRP (mg/dL)	13.54 (1.1–52.16)	7.5(1.1–16.3)	11.5(8.2–46.3)	16.5(8.1–36.3)	18.5(9.1–52.16)	0.01
D-Dimer (mg/L)	0.95 (0.27–33.2)	0.71(0.28–9.3)	0.99 (0.3–15.3)	1.1 (1.8–7.3)	2.3(1.21–13.3)	< 0.05
Leucocytes (10^3^ µL)	5.91 (2.1–19.12)	4.5(3.3–17.2)	8.5 (2.1–12.2)	7.5(5.4–19.12)	11.5(7.3–12.2)	0.56
S creatinine (mg/dL)	0.87 (0.50–8.26)	0.75(0.50–1.4)	0.81(0.61–1.9)	1.5 (0.80–8.2)	2.15 (0.50–1.4)	0.01
Bilirubin (mg/dL)	0.40 (0.2–4.8)	0.3 (0.2–1.1)	1.1(0.4–2.1)	0.8 (1.2–3.1)	0.9 (1.4–4.8)	0.65
Co-morbidities						
Diabetes	166 (18.2%)	64 (50.39%)	31 (33.3%)	49 (68.05%)	13 (46.4%)	0.07
Hypertension	171 (18.7%)	62 (48.81%)	34 (36.95%)	57 (79.16%)	9 (32.1%)	0.08
Heart disease	49 (5.3%)	18 (14.17%)	16 (17.20%)	11 (15.27%)	4 (14.28%)	0.61
COPD /chest	43 (4.7%)	15 (11.81%)	12 (12.90%)	10 (13.8%)	6 (21.4%)	0.54
Neurological	31 (3.4%)	13 (10.23%)	9 (9.6%)	5 (6.9%)	4 (14.2%)	0.27

The mean age was higher for patients with unfavorable prognosis (*P* < 0.01). No gender predicts bad prognosis. No symptoms predicted prognosis except dyspnea which is mildly more common in patients with poor prognosis. On the other hand laboratory findings showed significant difference between patients with good and poor prognosis. The oxygen saturation SP O2 was significantly lower in patients with bad prognosis than patients with good prognosis (*P* value 0.01). The mean CRP, D-Dimer and S creatinine were significantly higher in patients with poor prognosis (*P* values 0.01, < 0.05 and 0.01, respectively). No co-morbidities were associated with poor prognosis in the current study.

### CT chest findings

In the 910 patients initially studied for Covid-19 pneumonia, most lesions were bilateral (808 patients, 88.8%), most patients in all groups were bilateral, with no significant difference between the patients with good prognosis and poor prognosis. Most patients involved in the study had lesions in three lobes or more, with only 179/910 (19.6%) having lesions in one or two lobes. Patients with un-favorable prognosis tend to have more lobe involved than patients with good prognosis with significant difference (*P* = 0.01) (Table 2).

**Table 2:** Chest CT findings in all patients

	All patients (910)	Stable patients (127/320)	Hospitalized stable patients (93/320)	Patients needed ICU (72/320)	Died (28/320)	*P* value
**Distribution**						0.71
Bilateral	808 (88.8%)	104 (81.8%)	81(87.1%)	69 (95.8%)	28(100%)	
Unilateral	102 (11.2%)	23 (18.1%)	12(12.9%)	3 (4.1%)	(0%)	
**No of segments involved**						0.01
One	43 (4.7%)	13 (10.2%)	5(5.3%)	0(0%)	0(0%)	
Two	136 (14.9%)	22(17.3%)	7(7.5%)	3(4.1.1%)	0(0%)	
Three	198 (21.7%)	24(18.9%)	12(12.9%)	6 (8.3%)	2(7.1%)	
Four	292 (32.1%)	46(36.2%)	36(38.7%)	11(15.2%)	5(17.8%)	
Five	246 (27.04%)	22(17.3%)	33(35.4%)	53(73.6%)	21(75%)	
**Shape**						
Diffuse	124 (13.6%)	21 (16.5%)	23(24.7%)	28(38.4%)	11(42.8%)	0.01
Peripheral	89 (9.7%)	17 (13.3%)	22(23.6%)	20(27.4%)	6 (21.4%)	0.53
Peripheral and central	30 (3.2%)	4(3.1%)	1 (1.1%)	7(9.6%)	4 (14.2%)	0.02
Central	5 (0.05%)	− (0%)	− (0%)	1(1.3%)	1 (3.5%)	0.01
Patchy	532 (58.4%)	63(49.6%)	42(45.1%)	38(52.7%)	16(57.1%)	0.54
Nodular	123 (13.5%)	26(20.7%)	13(13.9%)	1(1.3%)	− (0%)	0.001
Mixed	131 (14.3%)	17(13.3%)	15(16.1%)	5 (6.9%)	1(3.5%)	0.01
**Density**						
GGO	512 (56.2%)	96(75.5%)	44(58.1%)	32(43.8%)	11(39.3%)	0.081
Consolidation	178 (19.5%)	4 (3.1%)	10(10.7%)	9(12.3%)	8(28.6%)	0.03
Mixed	220 (24.1%)	23(18.1%)	29(31.2%)	31(42.5%)	9(32.1%)	< 0.05

**Fig. 1:**
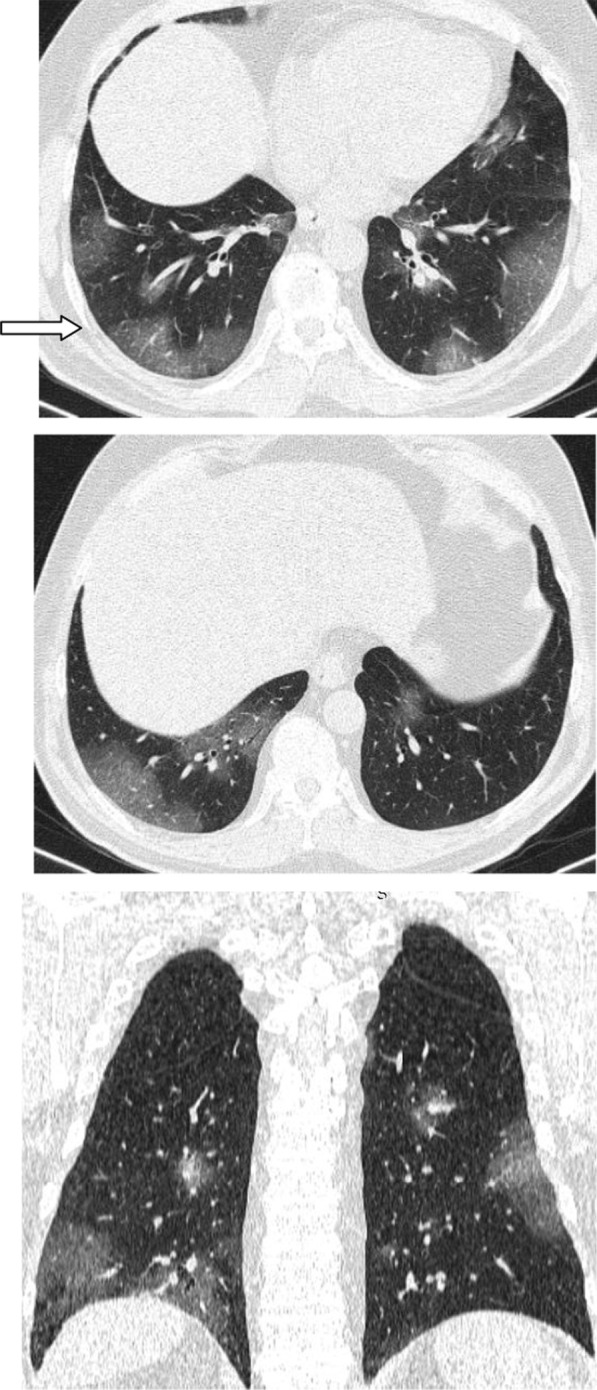
Male patient, age 34 years, with pure ground glass opacities scattered in both lungs. All lesions showed patchy shape

**Fig. 2:**
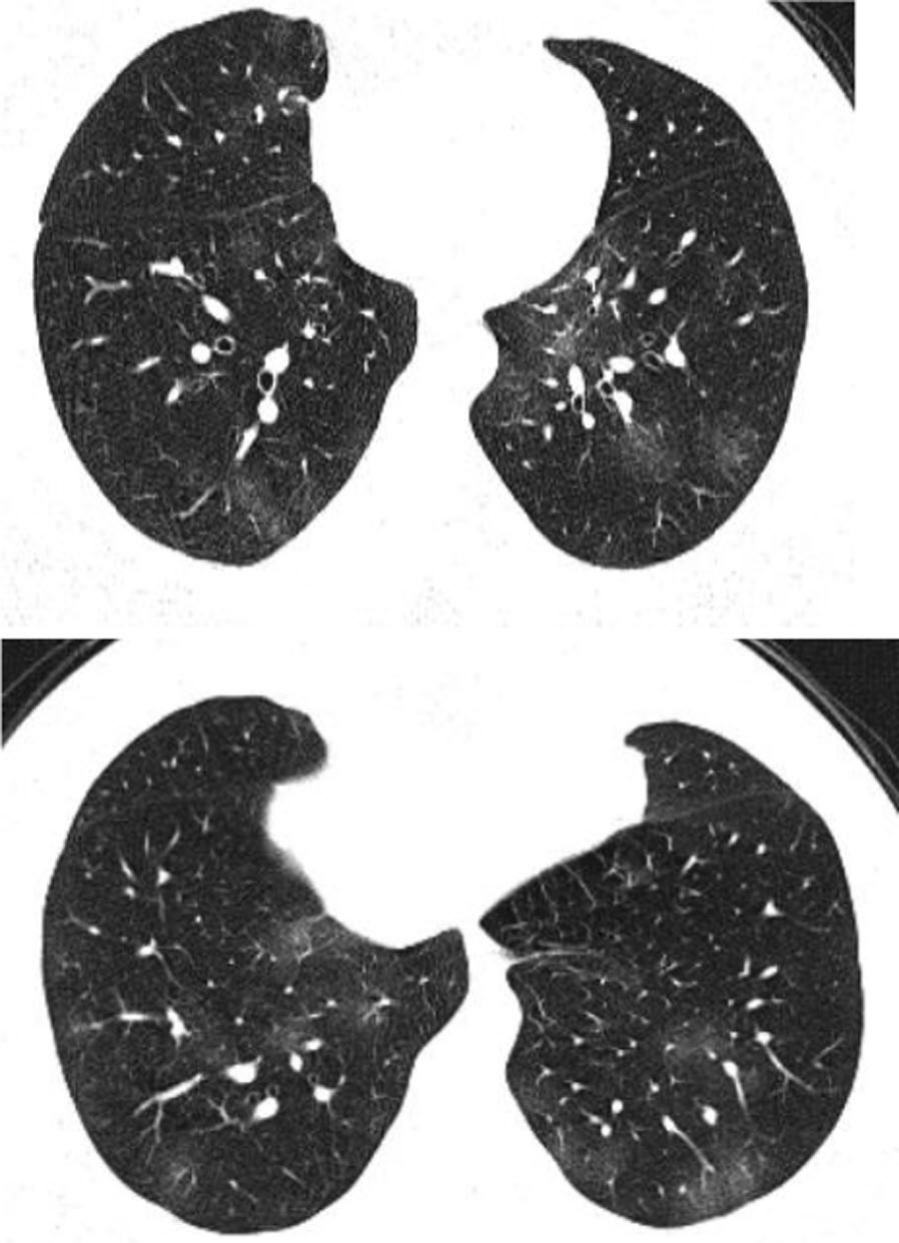
Male patient, aged 55 years, with bilateral central and peripheral pure ground glass infiltration with patchy shape

**Fig. 3:**
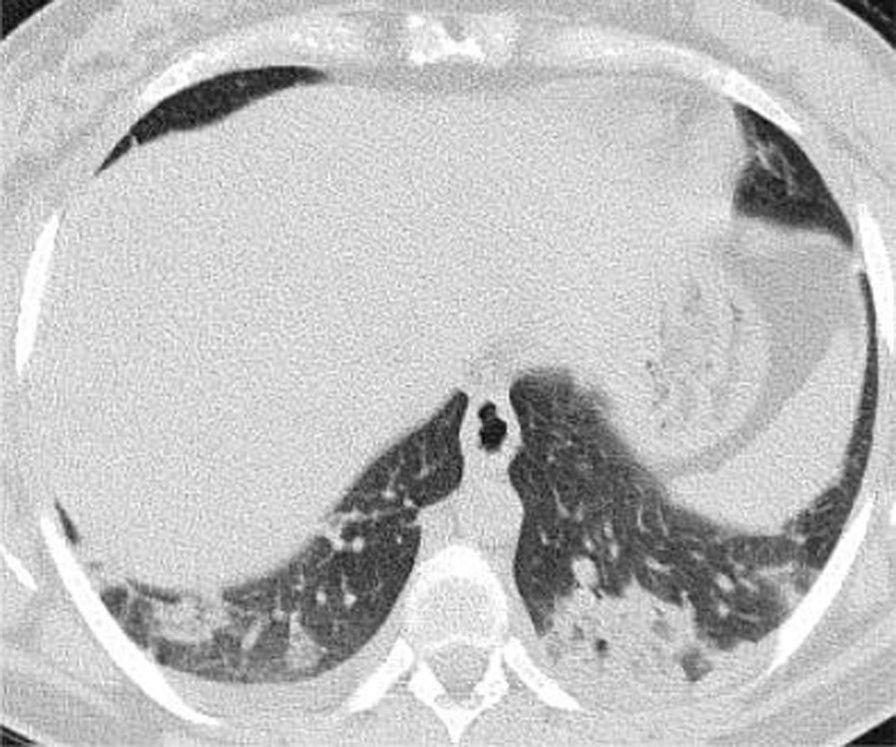
Female patient, aged 35 years, after 7 days from onset of fever. Bilateral lower lobes patchy areas of consolidation with air bronchogram and mild right pleural effusion

**Fig. 4:**
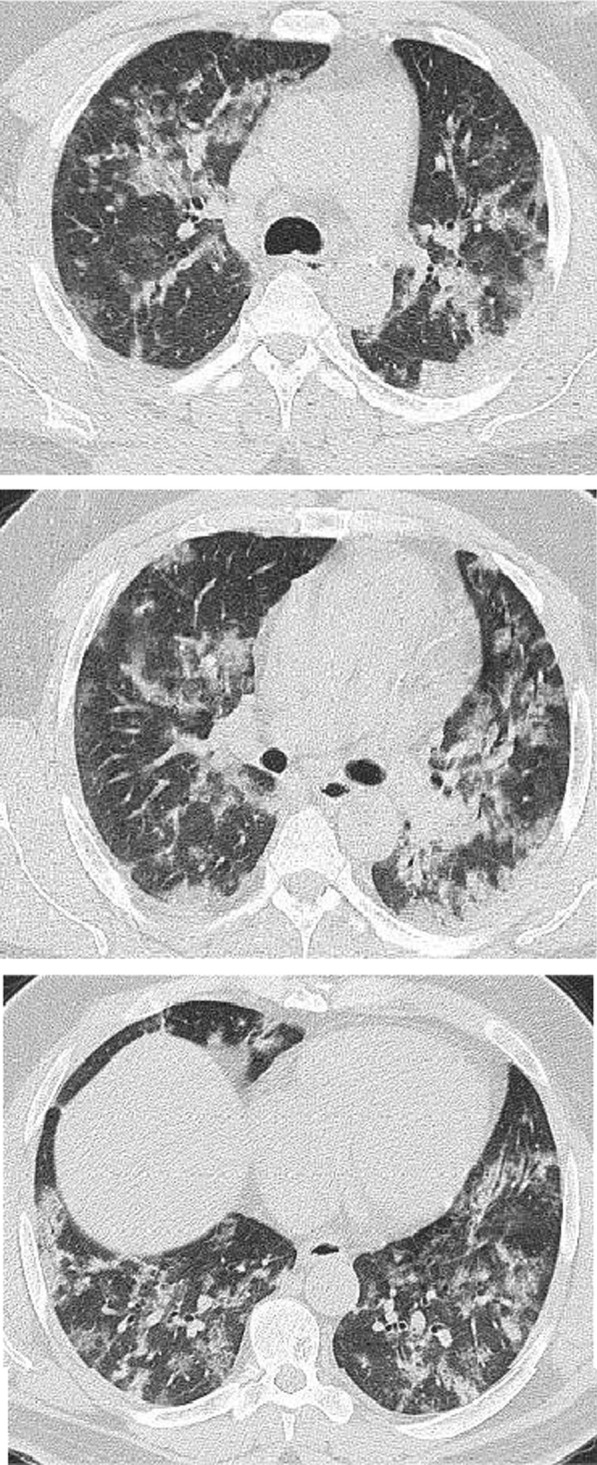
Male patient aged 57 years, 11 days after the onset of symptoms. Mixed patchy consolidation and ground glass infiltration

**Fig. 5:**
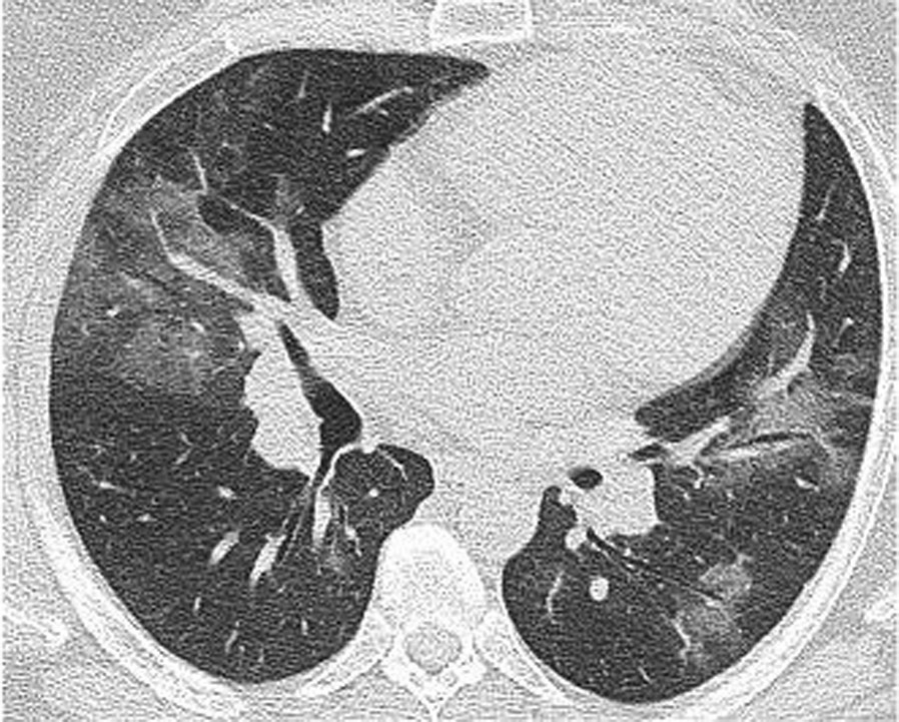
Male patient, aged 62 years four days from onset of symptoms. Wide patches of ground glass infiltration and dense large vessels

**Fig. 6:**
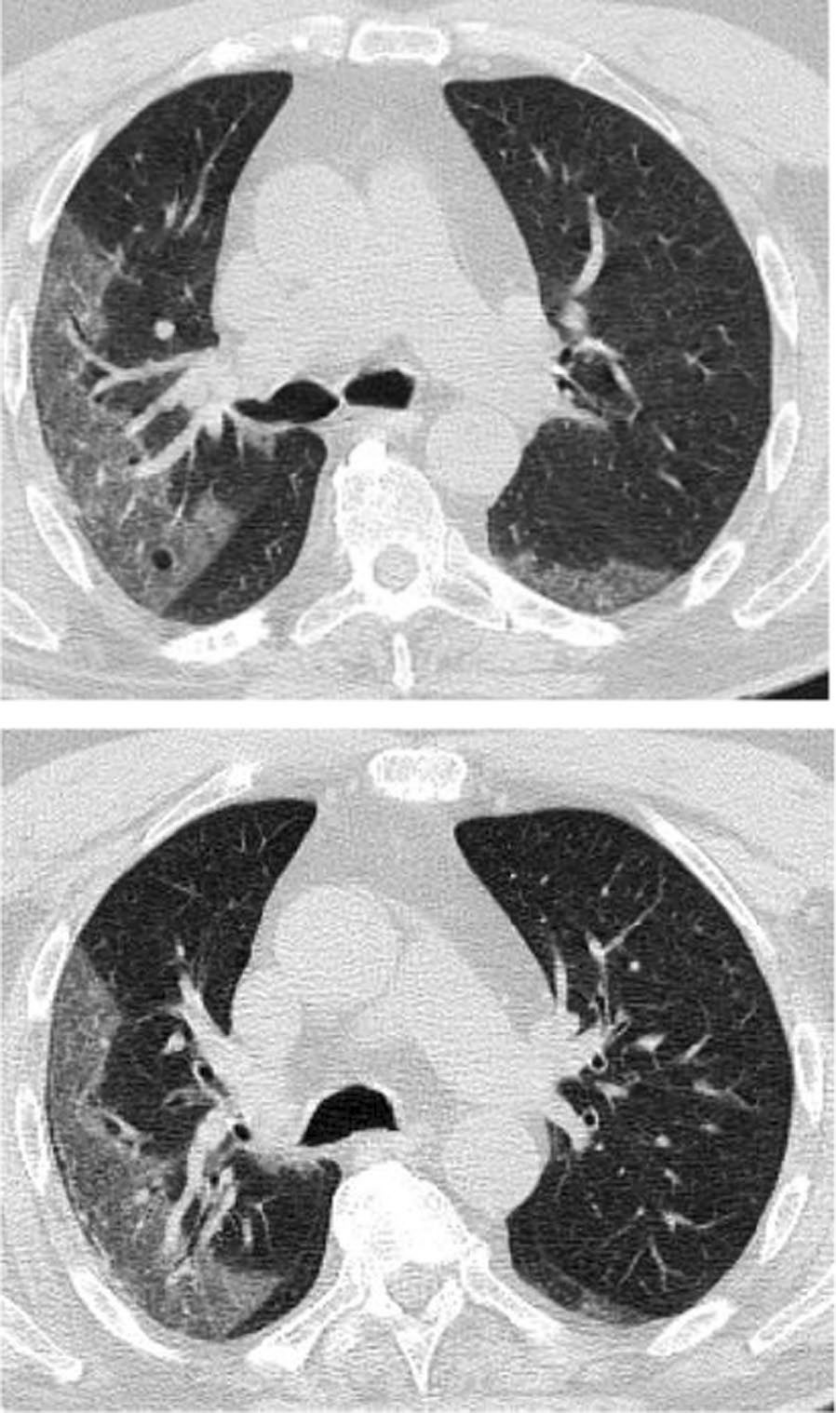
Male patient aged 43 years, with diffuse peripheral ground glass infiltration with dense and large vessels

**Fig. 7:**
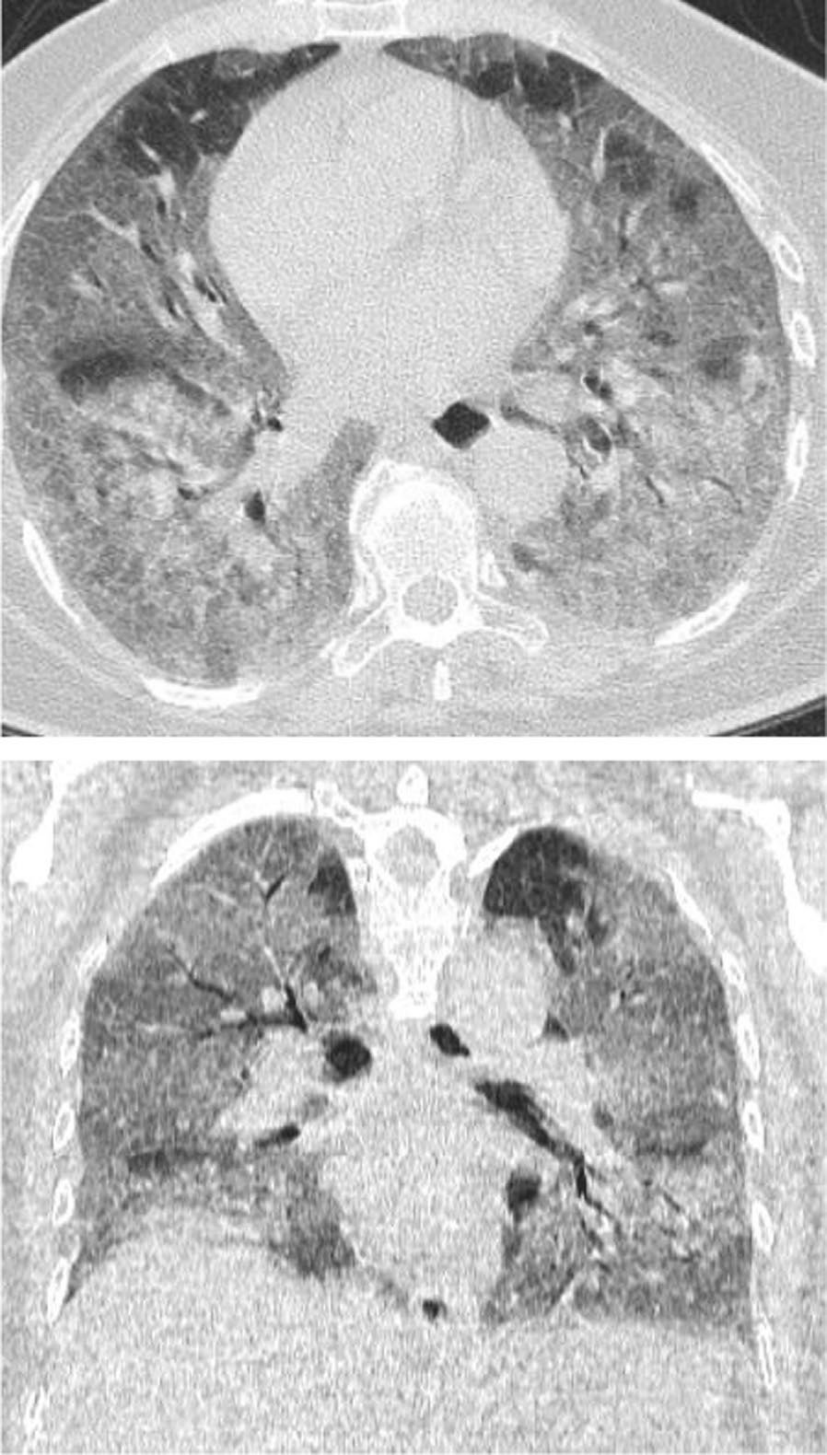
Female patient aged 71 years, with fever, dyspnea and neurological manifestations. Diffuse central and peripheral ground glass infiltration mounting to whitening out of the lung parenchyma. The CT severity score was 23

**Fig. 8:**
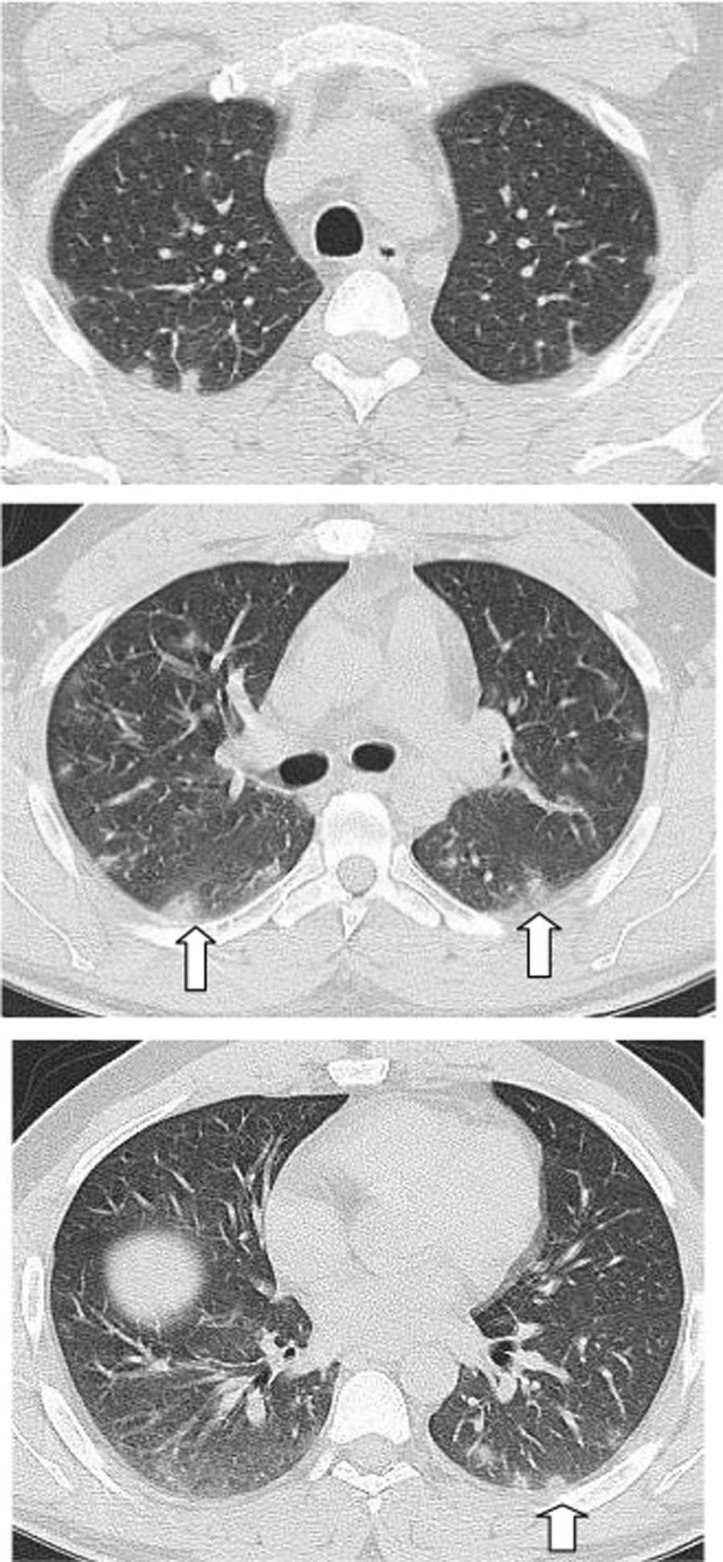
Male patient 22 years, 3 days after the onset of symptoms. Multiple peripheral subpleural nodules, with no cavitation or cystic changes. Patient received treatment at home

**Fig. 9:**
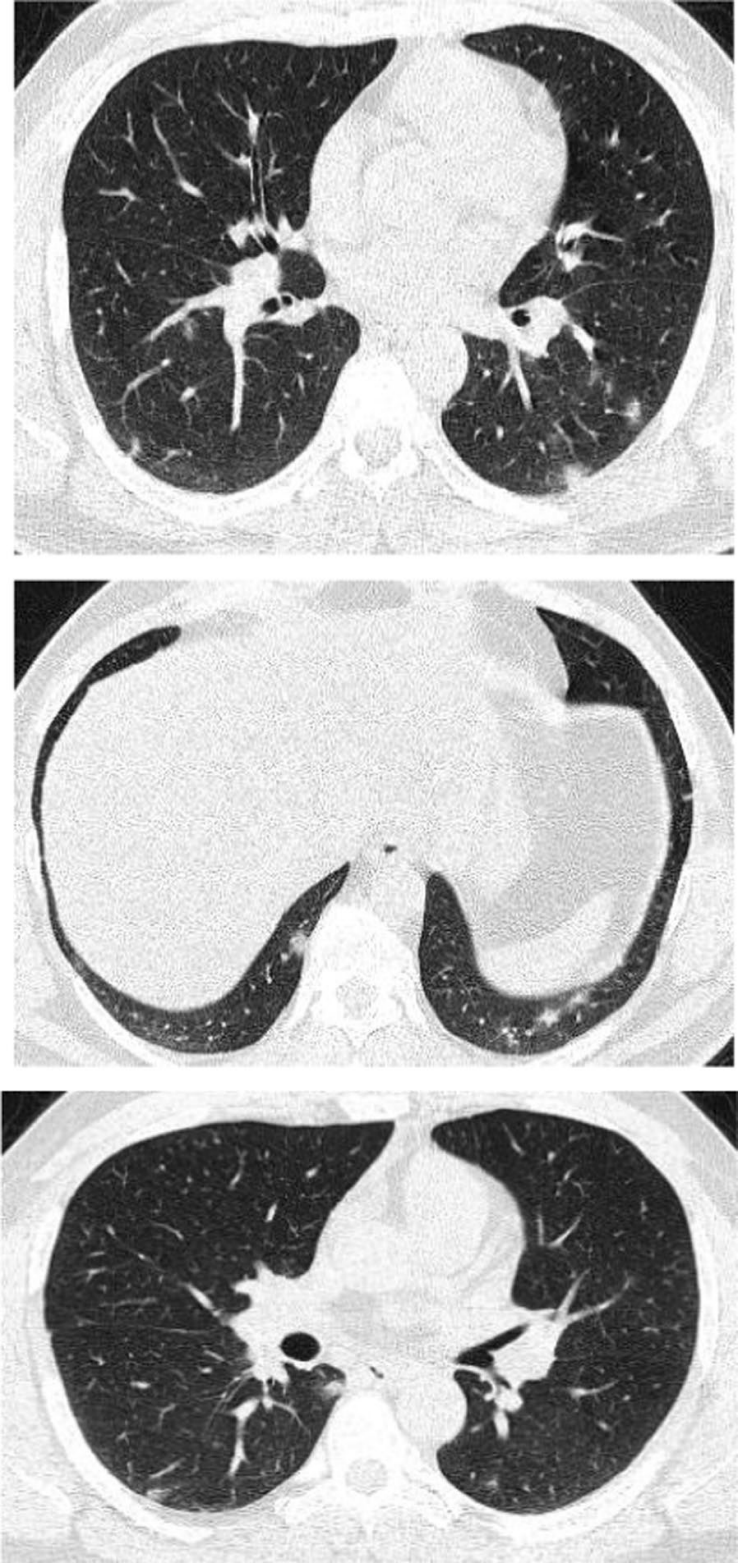
Male patient, aged 35 years, with fever for 6 days. Multiple small nodular shadows in both lungs, predominantly subpleural

**Fig. 10:**
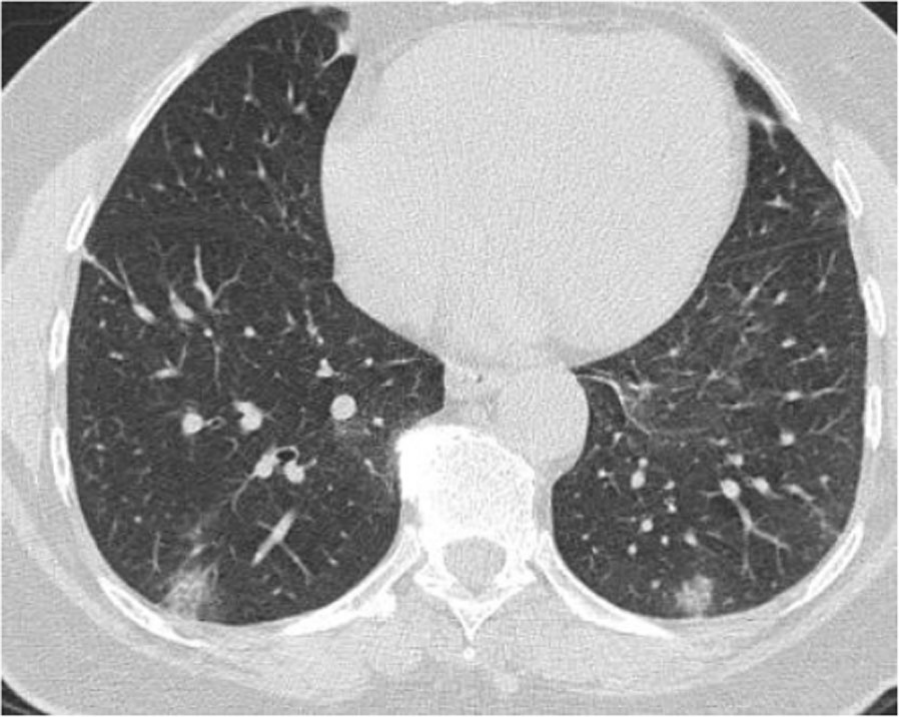
Male patient, aged 28 years, Nodular shape of the lesions. The nodular pattern carried a good prognosis in the current study

**Fig. 11:**
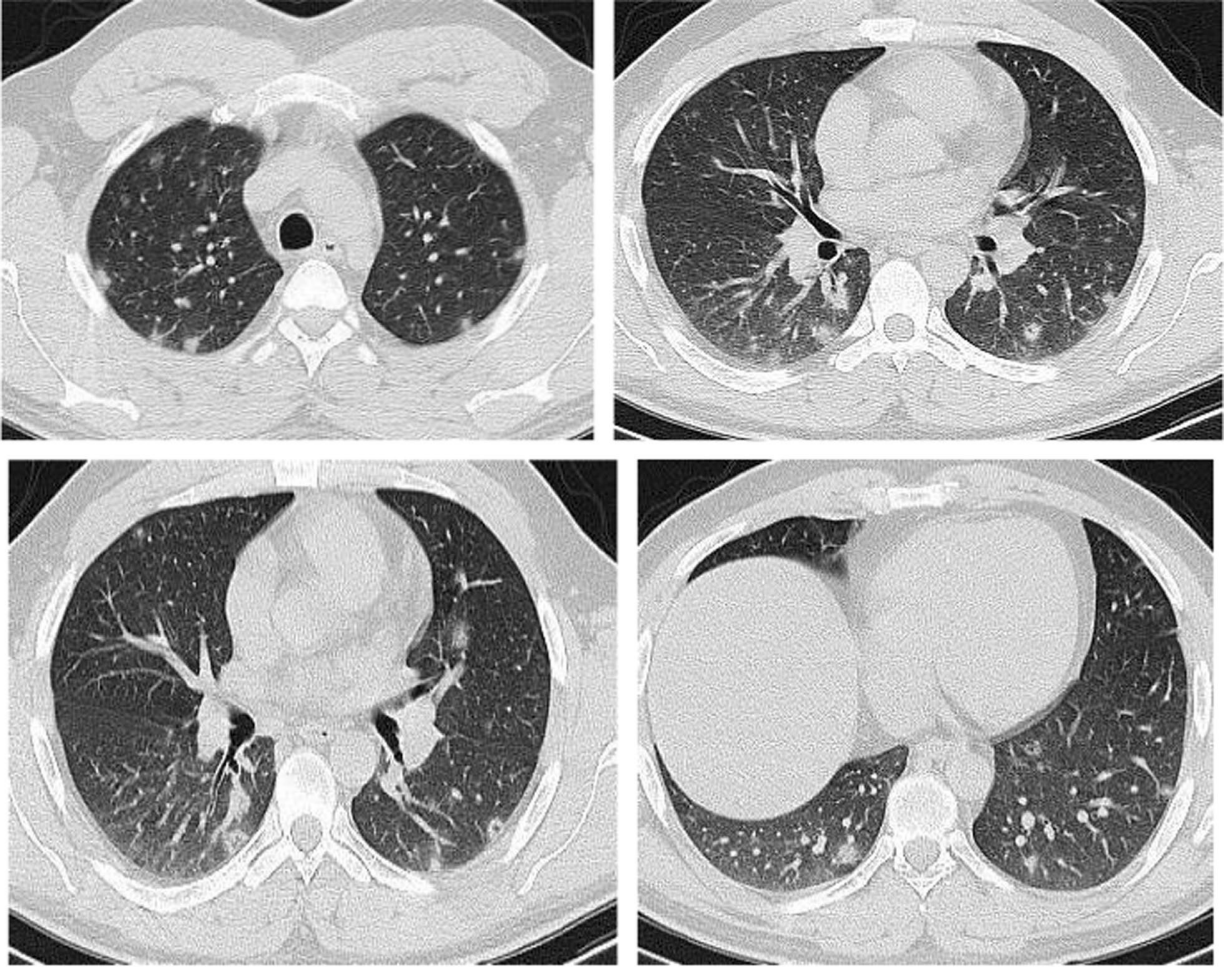
Male patients, 23 years old, with fever and cough for 4 days. Predominantly nodular pattern, with small cavitary lesions/cystic changes

**Fig. 12:**
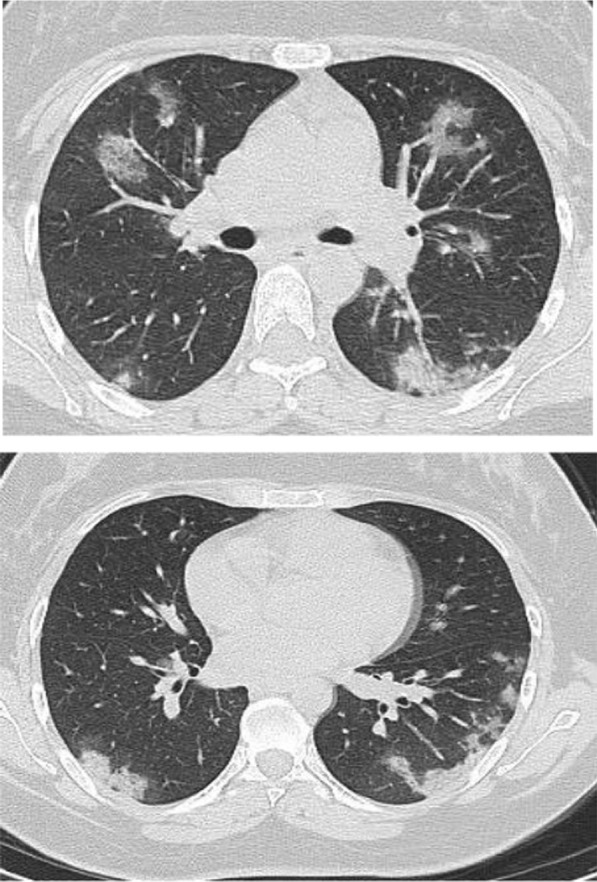
Female patient, aged 27 years, with multiple patches of consolidation with mixed nodular and patchy shape

Regarding the shape and pattern of the lesions, the patchy pattern (Figs. 1, 2, 3, 4, 5) was the most common, found in 532/910 patients (58.4%), the nodular pattern was the least common 123/910 (13.5%). The diffuse pattern was reported in 124 (13.6%). The diffuse pattern is generally associated with poor prognosis (Figs. 6, 7), especially if the central zones are involved (Table 2). Interestingly, patients with nodular pattern either alone or associated with patchy pattern (mixed pattern) had a good prognosis (Figs. 8, 9, 10, 11, 12), with statistically significant difference (0.001 and 0.01). The patchy pattern had no significant difference between the patients with good prognosis and patients with poor prognosis (*P* = 0.54).

The ground glass density was the most common reported density in the study 512/910 (56.2%), but it was present in the good and poor prognosis, with no significant difference. On the other hand, the consolidation either if present alone or associated with ground glass opacity was associated with poor prognosis with statistically significant difference (0.03 and < 0.05, respectively).

**Fig. 13:**
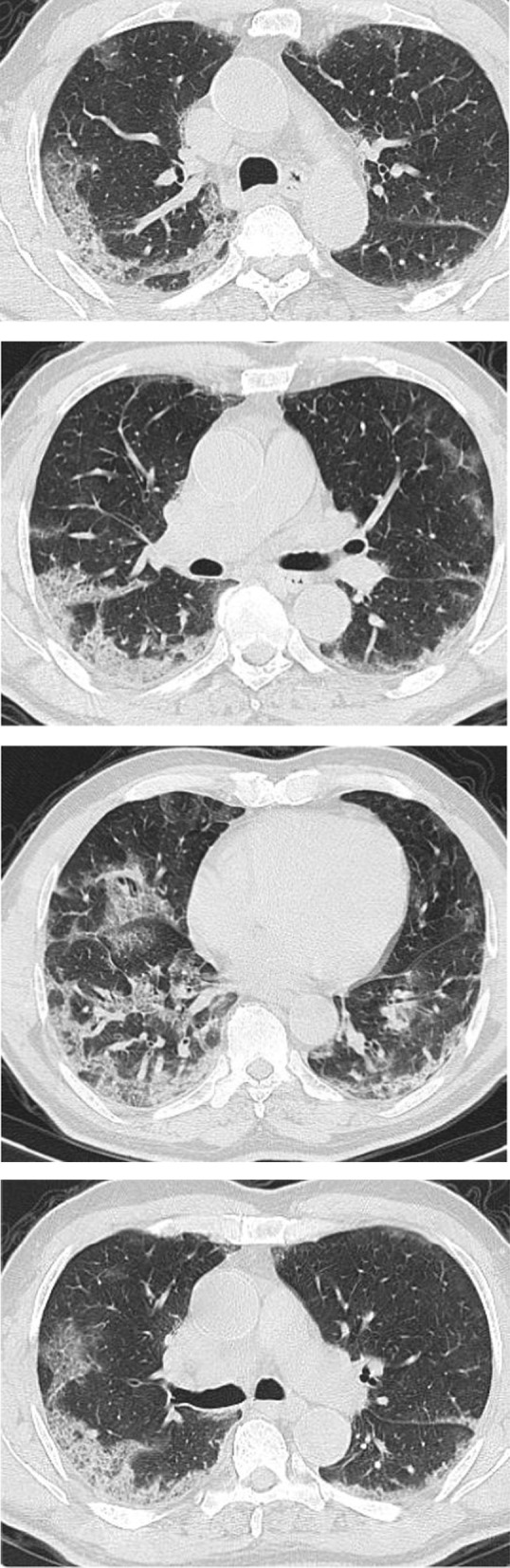
Male patient aged 32 years, with wide areas of consolidation mainly peripheral and subpleural, with crazy pavement appearance. This pattern associated with high CT-SS carried unfavorable prognosis in the current study

Multiple associated signs were reported. The halo sign and reversed halo were reported in 12.8% and 10.1% of the patients included in the study, respectively. Both signs were reported more frequently in patients with good prognosis, with statistically significant difference (*P* value 0.001). Also the transverse sub-pleural lines were associated with good prognosis. The transverse sub-pleural lines were a relatively common sign in the current study, reported in 317(34.8%) of the involved patients. It was reported in 71/220 (32.3%) patients with favorable prognosis and only 7/100 (7%) of patients with unfavorable prognosis, with statistically significant difference (*P* value 0.01). The crazy pavement sign (Fig. 13) reported in 376(41.3%) of the involved patients. The crazy pavement sign was reported more frequently in patients required hospitalization or ICU and was reported in 53 (56.9%) of patients required hospitalization, and it was reported in 11 (39.2%) deceased patients. Also air bronchogram was reported more frequently in patients with poor prognosis than patients with good prognosis (16/100; 16% Vs 12/220; 5.4%) with statistically significant difference. The pleural effusion, pericardial effusion and pneumo-mediastinum were associated with poor prognosis (Table 3).

**Table 3:** Associated CT signs in all patients

	All patients (910)	Stable patients (127/320)	Hospitalized stable patients (93/320)	Patients needed ICU (72/320)	Died (28/320)	*P* value
Air bronchogram	134 (14.7%)	8 (6.2%)	4 (4.3%)	19 (26.3%)	7 (25%)	< 0.01
Halo sign	117 (12.8%)	17 (13.3%)	18 (19.3%)	3 (4.1%)	− (0%)	0.01
Reversed halo sign	92 (10.1%)	19 (14.9%)	21 (22.6%)	8 (10.9%)	1 (3.5%)	0.02
Crazy Pavement pattern	376(41.3%)	22 (17.3%)	53 (56.9%)	29(40.2%)	11 (39.2%)	0.002
Cavitation	72 (7.9%)	11 (8.6%)	14 (3.3%)	7 (9.7%)	3 (10.7%)	0.51
Bronchiectasis	29 (3.1%)	1 (0.07%)	− (0%)	2 (2.7%)	2 (7.1%)	0.34
Pleural effusion	37 (4.06%)	− (0%)	− (0%)	2 (2.7%)	2(7.1%)	0.01
Pericardial effusion	11 (1.2%)	− (0%)	1 (0.9%)	1 (1.3%)	2(7.1%)	0.051
Transverse subpleural lines	317(34.8%)	43 (33.8%)	29 (31.1%)	6 (8.3%)	1 (3.5%)	0.01
Large vessel sign	354 (38.9%)	38 (29.9%)	21 (22.6%)	12 (16.6%)	9 (32.1%)	0.51
Dense vessel sign	169 (18.6%)	23 (18.1%)	12 (12.9%)	21 (29.1%)	11(39.2%)	0.05
Pneumomediastinum	9 (0.09%)	0(0%)	− (0%)	1 (1.3%)	2(7.1%)	0.01
Pneumothorax	6 (0.06%)	0(0%)	− (0%)	1(1.3%)	2(7.1%)	0.01
Mediastinal lymphadenopathy	71(7.8%)	1(0.07%)	− (0%)	2(2.7%)	3(10.7%)	

**Fig. 14:**
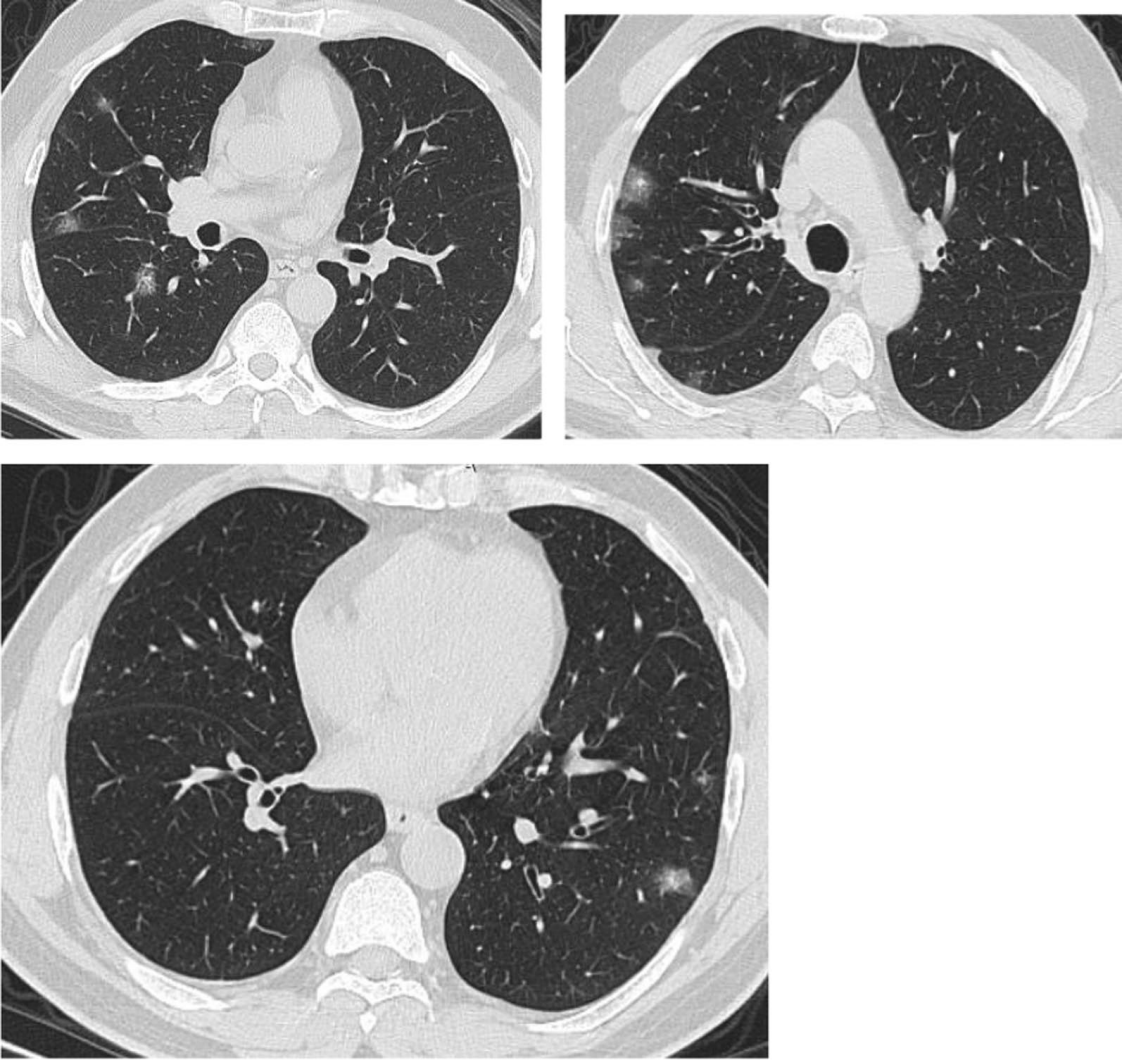
Female patient aged 21 years, with multiple nodular lesions and halo sign

**Fig. 15:**
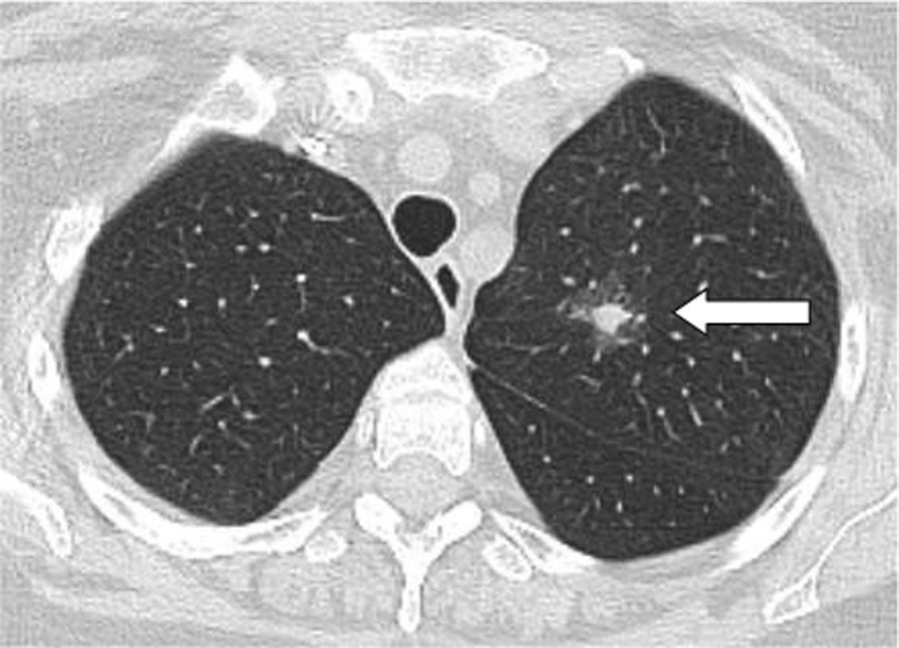
Female patient aged 47 years, with single nodule with Halo sign

**Fig. 16:**
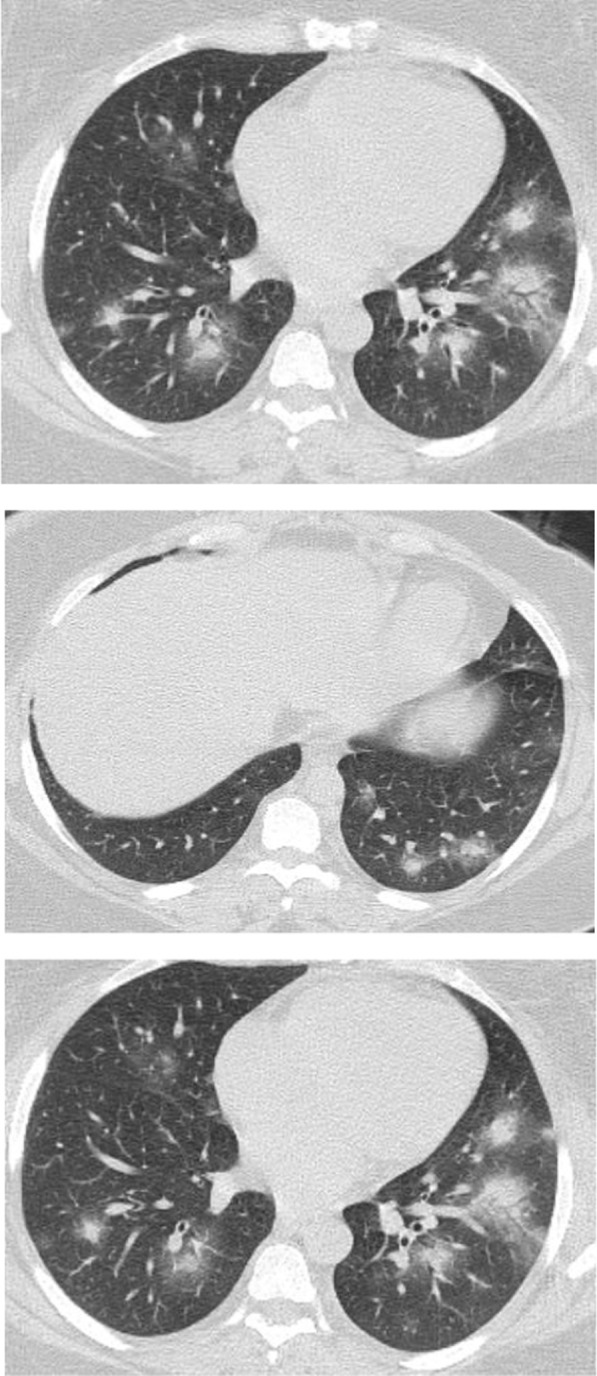
Male patient, aged 25 years, with halo sign 7 days after onset of symptoms, with good prognosis

**Fig. 17:**
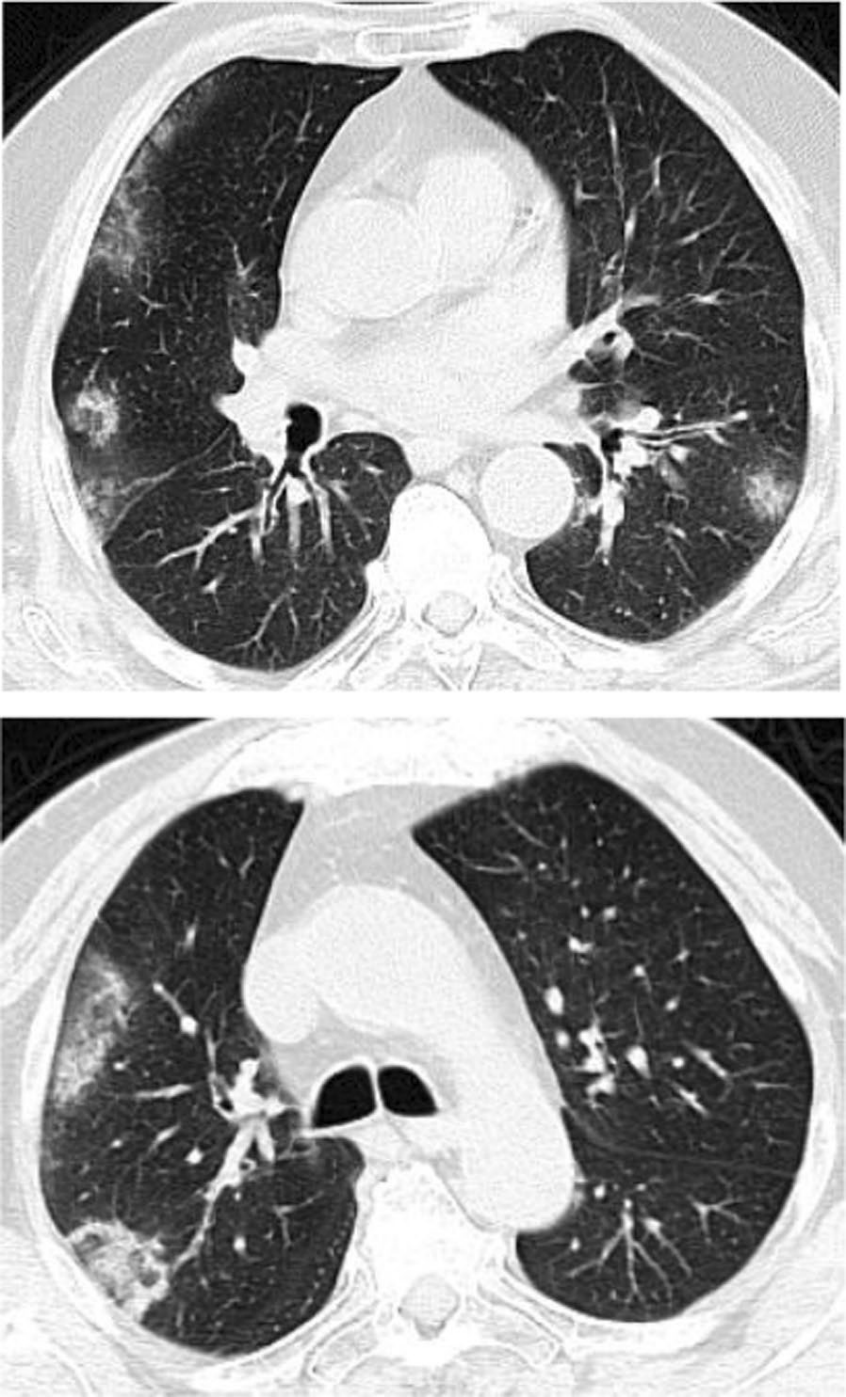
Male patient, aged 32 years, with multiple subpleural patches with reversed halo sign. Most lesions were nodular in shape. This pattern carried a good prognosis in the current study

**Fig. 18:**
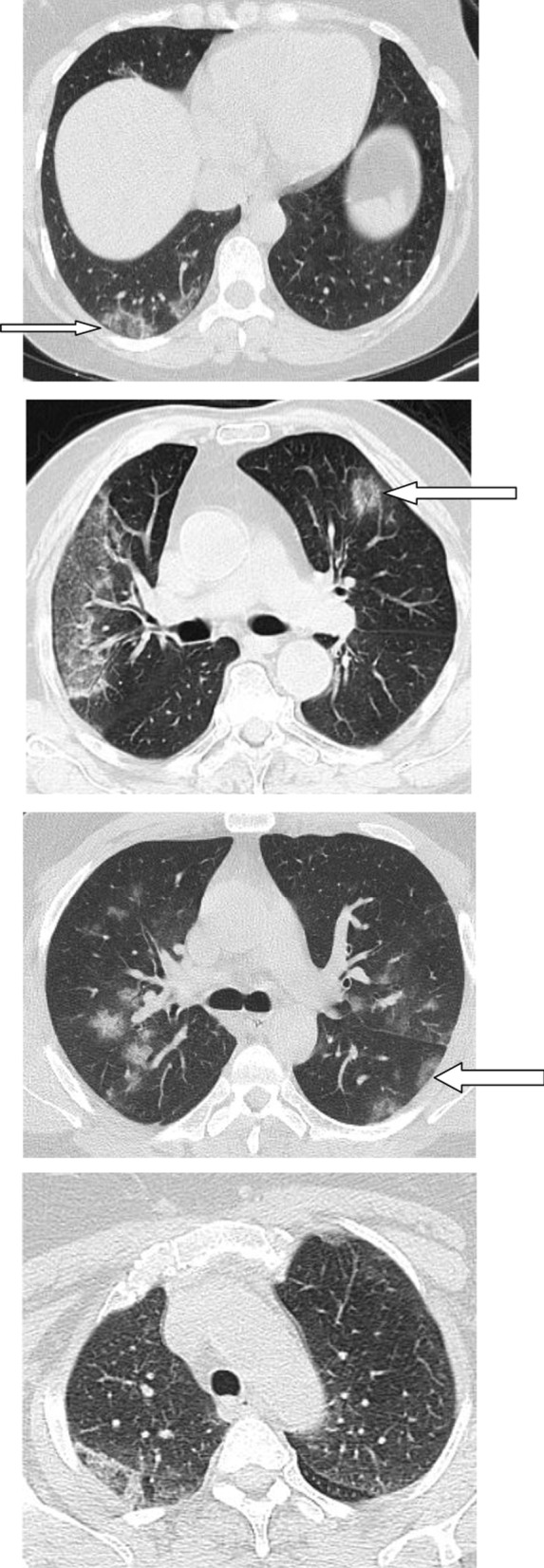
Reversed halo sign in four different patients

**Fig. 19:**
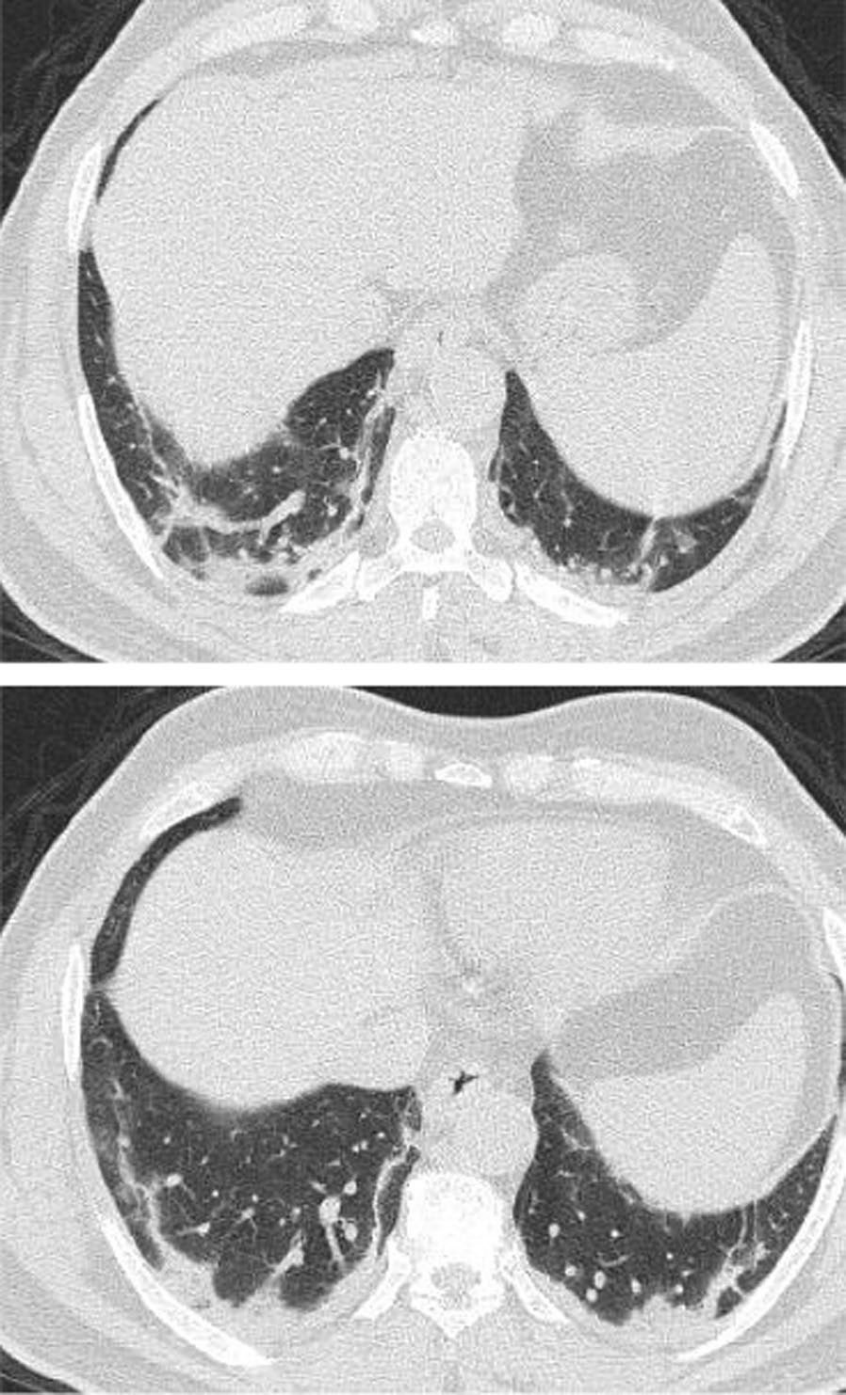
Male patient aged 34 years, with multiple subpleural dense lesions with transverse subpleural lines

**Fig. 20:**
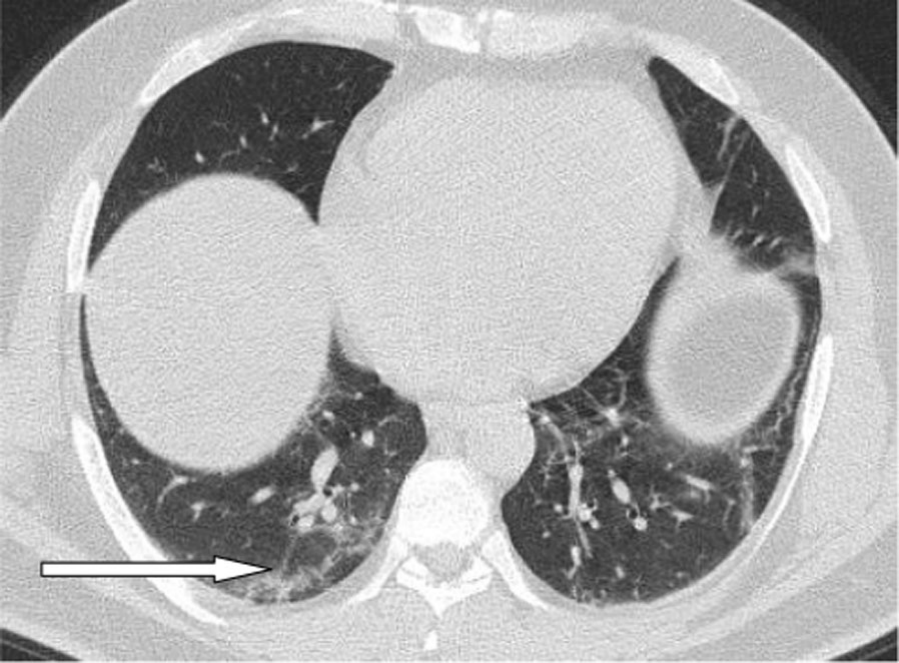
Female patient, 32 years old with sub-pleural lines, peripheral and posterior, with subpleural transparent line

The mean CT severity score for patients with poor prognosis was 15.2. The mean CT severity score for patients with good prognosis was 8.7., with statistically significant difference (*P* = 0.001). In the current study among the 320 patients we could follow their outcome, 147 had mild CT severity score (1–8), of them only 29 (19.7%) had unfavorable prognosis and only 5 patient died with initial CT severity score < 8. The patients with moderate CT severity score had no statistically significant difference between the patient's with good and poor prognosis. Most patients with severe CT severity score had unfavorable prognosis (38/59 patients; 64.4%). Of the 28 patients died in this study, 5(17.8%) had initial score 1–8, 8 patients (28.5%) had score 9–17 and 15 patients (53.3%) had score > 17. On the other hand of the 127 patients with mild symptoms and treated at home 73 patients (57.5%) had score 1–8; 49 patients (38.6%) had score 9–17 and only 5 patients (3.9%) had score > 17 (Figs. 14, 15, 16, 17, 18, 19, 20).

## Discussion

COVID-19 is potentially fatal and highly contagious disease, both factors making it a significant public health problem. Early diagnosis and isolation are important in controlling the disease spread. In the current study we described the CT findings in 910 Egyptian patients [35] (Table 4).

**Table 4:** Distribution of CT severity score among all patients

	All patients (910)	Stable patients (127/320)	Hospitalized stable patients (93/320)	Patients needed ICU (72/320)	Died (28/320)	*P* value
Score 1–8	329(36.2%)	73(57.5%)	41(44.1%)	14(19.4%)	5(17.8%)	< 0.01
Score 9–17	417(45.8%)	49(38.6%)	36(33.5%)	35(48.6%)	8(28.5%)	0.15
Score 18–25	164(18.02%)	5(3.9%)	16(14.8%)	23(31.9%)	15(53.3%)	< 0.001

In the current study, patients with older age, and patients with diabetes or hypertension had more severe clinical disease than younger patients and patients with co-morbidities. Also, patients with severe disease suffered from dyspnea more frequently than patients with mild forms. Patients with severe disease had different laboratory investigations especially higher CRP, D-Dimer and S-creatinine. The presence of dyspnea may indicate more damage of the alveoli or the presence of interstitial inflammatory response. High Serum CRP was found to be associated with poor outcome because it indicates diffuse and severe inflammatory reaction [36]. Also the increase in serum D-dimer was supposed to be mortality predictor as a consequence of disseminated coagulopathy [37].

The great majority of our patients had bilateral disease (88.8%) and the abnormalities involved three segments or more (80.8%). Our results are approximate to the meta-analysis of Garg et al. [38], with reported incidence of unilateral disease in 286/3141 patients (9.1%). Also, Adams et al. [22], in a meta-analysis including 3466 patients reported pooled prevalence of unilateral disease in 15% of the patients. In the current study, the prevalence of ground glass opacity, ground glass opacity and consolidation and consolidation only were 56.2%, 24.1% and 19.5%, respectively, the prevalence and order are similar to previous reports from different countries [22, 38, 39].

Ground glass opacity is the earliest and most consistent sign in COVID-19 pneumonia. In literature, its incidence ranged from 65%–100% [6, 7, 40]. The pathogenesis of GGO is presumed to be alveolar edema, alveolar exudate and hyaline membrane formation [41]. In the current study the general incidence of GGO was 80.5%, because we included patients with early and late disease. We found no significant difference in the incidence of GGO between patients with favorable and un-favorable prognosis.

Consolidations either alone or with GGO reported in 43.6% of patients in the current study. Consolidations were reported in 47% of patients in the study by Wong et al. [42]. Zhou et al. [43] in a study involved 100 patients with duration of 1–7 days from the onset of symptoms reported GGO and consolidations in 43% of patients. In another study Pan et al. [7] reported that the incidence of consolidation increases with the duration of symptoms. In the current study, consolidations either alone or with GGO were observed more frequently in patients with un-favorable prognosis with statistically significant difference. In a recent study, Zhan et al. [44] reported consolidation in 4.9% of mild pneumonia cases and 70.1% of cases with severe pneumonia (*P* < 0.017).

Different shapes of the lesions were reported in our study, we found the rounded shape either alone or mixed with patchy shape were reported more frequently in stable patients with favorable prognosis, Diffuse pattern especially if central was associated with poor prognosis. Tabatabaei et al. [31] reported nodular shape in 26% of patients with nodular patients, and 8% of deceased patients, while the central involvement was reported in 85% of deceased patients and in 56% of patients needed inward hospitalization. Also Parry et al. [45] reported central lung involvement in 85% in unstable patients, with statistically significant difference with the stable group (*P* < 0.05%).

The crazy paving sign was reported in 41.3% of patients included in this study, and generally was reported in patients needed hospitalization or ICU. The crazy paving sign is presumed to reflect alveolar edema and interstitial inflammation, relatively a specific sign of COVID 19 pneumonia and reported in as high as 92% of cases [46, 47]. The initial reports suggested that crazy paving pattern is a sign of progressive disease [48, 49]. Zahan et al. [44], reported the incidence of localized crazy paving sign in 65% of patients with mild pneumonia cases and in 23.4% of the severe cases (*P* < 0.001). Meiler et al. [50] reported crazy paving sign as the only independent predictor of poor outcome by multivariable analysis and also predicted negative outcome by univariable analysis, and they suggested a correlation between its presence and the incidence of pulmonary edema and acute respiratory distress syndrome. Tabatabaei et al. [31] in a study included 120 patients noticed crazy paving sign more frequently in patients admitted to ICU and dead patients (*P* < 0.05).

The differential diagnosis of the halo sign [14–16] includes hemorrhagic nodules, fungal infection, metastasis, and viral infection [51]. The halo sign was reported in 12.8% of patients in the current study. The incidence in the literature ranged from 1.7–41.8% (with pooled incidence 23.7%) [38]. Though the halo sign reflects alveolar edema, hemorrhage or microcirculation thrombosis, in the current study, halo signs were associated with good prognosis.

The reversed halo sign was reported in 10.1% of patients included in the study. The reversed halo sign was not reported in any case with severe pneumonia in the study by Zhan et al. [44]. Parry et al. [45] reported reversed halo sign in 21% of patients with mild disease and in only 5% of patients with severe disease, but the difference was statistically insignificant. In the current study, the reversed halo sign was more common in patients with favorable diagnosis than patients with unfavorable diagnosis and the difference was statistically significant probably because this sign reflects partial clearing of the lesions leaving an area of low attenuation.

The sub-pleural lines were reported frequently in the current study (317 patients, 34.8%) of patients included in the study. The reported incidence in literature is 17–28% [33]. The sub-pleural lines were reported more frequently in patients with good prognosis, with statistically significant difference (0.01). The appearance of sub-pleural lines may indicate triggering of reparative process, and most likely fibrotic in nature [52]. Li et al. [53] reported sub-pleural lines more frequently in patients with severe disease, but the difference was statistically insignificant.

We reported two signs in the pulmonary vessels in the current study, the large vessel sign and the dense vessel sign. The large vessel sign showed no significant difference between patients with favorable and unfavorable prognosis, while the dense vessel sign was reported more in patients with unfavorable prognosis, with statistically significant difference. The vascular enlargement sign results from injury of capillary endothelial cells and alveolar epithelial cells. The dense vessel sign is probably related to increased blood viscosity or localized arterial thrombosis which results from inflammatory cytokines. The dense vessel sign may warrant the use of anticoagulation or the increase in its dose. Ackermann et al. [54] reported vascular abnormalities in patients died from COVID-19 pneumonia including localized thrombosis and microangiopathy.

In the current study, most patients with un-favorable prognosis had high CT severity score (38% of patients had score > 18, and 43% had score 9–17). Our results are consistent with previous reports [9, 55, 56]. Xie et al. [55], reported higher CT severity score in critically ill patients than patients with mild symptoms. The CT-SS is probably a good indicator of disease severity and may provide a semi-quantitative measure of disease progression. Fancone et al. [56] in 130 symptomatic patients found CT SS more than 18 highly predictive of mortality. They even found CT-SS more accurate and better predictable of short-term mortality than serum CRP and D-Dimer, and they concluded that the direct visualization of the extent of parenchymal or alveolar injury is more important than the non-specific biomarkers. Also Saeed et al. [57] reported a positive correlation between the CT severity score and the increase in D dimer, severity of lymphopenia, increase oxygen requirement and the death rate. All these changes reflect the direct alveolar damage by the virus and the vascular changes [57].

There were some limitations of the study. First, we didn’t correlate the CT findings with the duration of symptoms or onset of the disease. Second, we used only the CT findings in the initial CT scan, follow-up CT scans were not investigated and did not correlate with the disease progression. Third, we did not take in consideration the type and quality of medical treatment for each patient. Fourth, we did not perform multivariate statistical analysis.

## Conclusion

Our results confirm the important role of the initial CT findings in the prediction of clinical outcome and short-term prognosis. Some signs like subpleural lines, halo sign, reversed halo sign and nodular shape of the lesions predict mild disease and favorable prognosis. The crazy paving sign, dense vessel sign, consolidation, diffuse shape and high severity score predict more severe disease and probably warrant early hospitalization. The high severity score is most important in prediction of unfavorable prognosis. The nodular shape of the lesions is the most important predictor of good prognosis.

## Data Availability

Data are available upon reasonable request.

## Abbreviations

GGO: Ground glass opacity
CTSS: Computed tomography severity score
COVID-19: Corona virus disease 2019
RT-PCR: Reverse transcription polymerase chain reaction
